# Model-based clustering of multi-tissue gene expression data

**DOI:** 10.1093/bioinformatics/btz805

**Published:** 2019-11-05

**Authors:** Pau Erola, Johan L M Björkegren, Tom Michoel

**Affiliations:** 1 Division of Genetics and Genomics, The Roslin Institute, The University of Edinburgh, Midlothian EH25 9RG, UK; 2 MRC Integrative Epidemiology Unit, University of Bristol, Bristol BS8 2BN, UK; 3 Department of Genetics and Genomic Sciences, Institute of Genomics and Multiscale Biology, Icahn School of Medicine at Mount Sinai, New York, NY 10029, USA; 4 Integrated Cardio Metabolic Centre (ICMC), Karolinska Institutet, Huddinge 141 57, Sweden; 5 Computational Biology Unit, Department of Informatics, University of Bergen, Bergen N-5020, Norway

## Abstract

**Motivation:**

Recently, it has become feasible to generate large-scale, multi-tissue gene expression data, where expression profiles are obtained from multiple tissues or organs sampled from dozens to hundreds of individuals. When traditional clustering methods are applied to this type of data, important information is lost, because they either require all tissues to be analyzed independently, ignoring dependencies and similarities between tissues, or to merge tissues in a single, monolithic dataset, ignoring individual characteristics of tissues.

**Results:**

We developed a Bayesian model-based multi-tissue clustering algorithm, revamp, which can incorporate prior information on physiological tissue similarity, and which results in a set of clusters, each consisting of a core set of genes conserved across tissues as well as differential sets of genes specific to one or more subsets of tissues. Using data from seven vascular and metabolic tissues from over 100 individuals in the STockholm Atherosclerosis Gene Expression (STAGE) study, we demonstrate that multi-tissue clusters inferred by revamp are more enriched for tissue-dependent protein-protein interactions compared to alternative approaches. We further demonstrate that revamp results in easily interpretable multi-tissue gene expression associations to key coronary artery disease processes and clinical phenotypes in the STAGE individuals.

**Availability and implementation:**

Revamp is implemented in the Lemon-Tree software, available at https://github.com/eb00/lemon-tree

**Supplementary information:**

Supplementary data are available at *Bioinformatics* online.

## 1 Introduction

Clustering gene expression data into groups of genes sharing the same expression profile across multiple conditions remains one of the most important methods for reducing the dimensionality and complexity of large-scale microarray and RNA-sequencing datasets (Andreopoulos *et al.*, 2008; D’haeseleer, 2005; van Dam *et al.*, 2017). Coexpression clusters group functionally related genes together, and reveal how diverse biological processes and pathways respond to the underlying perturbation of the biological system of interest. Traditionally, clustering is performed by collecting data from multiple experimental treatments (Eisen *et al.*, 1998), time points (Spellman *et al.*, 1998), cell or tissue types (Freeman *et al.*, 2007), or genetically diverse individuals (Ghazalpour *et al.*, 2006) in a single data matrix from which meaningful patterns are extracted using any of a whole range of statistical and algorithmic approaches. More recently, it has become feasible to probe systems along two or more of these dimensions simultaneously. In particular, we are interested in multi-tissue data, where gene expression profiles are obtained from multiple tissues or organs sampled from dozens to hundreds of individuals (Foroughi Asl *et al.*, 2015; Franzén *et al.*, 2016; Fu *et al.*, 2012; Greenawalt *et al.*, 2011; Grundberg *et al.*, 2012; GTEx Consortium, 2017; Hägg *et al.*, 2009; Keller *et al.*, 2008). These data can potentially reveal the similarity and differences in (co)expression between tissues as well as the tissue-specific variation in (co)expression across individuals.

However, when traditional clustering methods are applied to this type of data, important information is lost. For instance, if each tissue-specific sub-dataset is clustered independently, the resulting sets of clusters will rarely align, and to compare clusters across tissues, one will be faced with the general problem of determining cluster preservation statistics (Langfelder *et al.*, 2011). If instead the data are concatenated ‘horizontally’ in a single gene-by-sample matrix, a common set of clusters will be found, but these will be biased heavily towards house-keeping processes that are coexpressed in all tissues. A potentially more promising approach is to concatenate data ‘vertically’ in a tissue-gene-by-individual matrix, where the entities being clustered are ‘tissue-genes’, the tissue-specific expression profiles of genes (Dobrin *et al.*, 2009; Talukdar *et al.*, 2016). However, in studies with a large number of tissues, the number of individuals with available data in *all* tissues is typically very small, i.e. a large number of samples will have to be discarded to obtain a tissue-gene-by-individual matrix without missing data.

Dedicated clustering algorithms for multi-tissue expression data are scarce and mostly based on using the higher-order generalized singular value decomposition or related matrix decomposition techniques to identify common and differential clusters across multiple conditions (Li *et al.*, 2011; Ponnapalli *et al.*, 2011; Xiao *et al.*, 2014). However, these methods either require that all tissues have the same number of one-to-one matching samples (Ponnapalli *et al.*, 2011), or that tissue-specific coexpression networks are reconstructed for each tissue separately as a preliminary step (Li *et al.*, 2011; Xiao *et al.*, 2014). Bayesian model-based clustering methods, which model the data as a whole using mixtures of probability distributions (Fraley and Raftery, 2002; Ickstadt *et al.*, 2017; Si *et al.*, 2014), are an attractive alternative approach for clustering multi-tissue data, because they would allow, at least in principle, to account for different noise levels and sample sizes in different tissues and to incorporate prior information on the relative similarity between certain tissues based on their known physiological function.

Here we present a novel statistical framework and inference algorithm for model-based clustering of multi-tissue gene expression data, which can incorporate prior information on tissue similarity, and which results in a set of clusters, each consisting of a core set of genes conserved across tissues as well as differential sets of genes specific to one or more subsets of tissues.

## 2 Materials and methods

### 2.1 Approach

In model-based clustering, a partitioning of genes into non-overlapping clusters parametrizes a probabilistic model from which the expression data is assumed to have been generated, typically in the form of a mixture distribution where each cluster corresponds to one mixture component. Using Bayes’ theorem, this can be recast as a probability distribution on the set of all possible clusterings parameterized by the expression data, from which maximum-likelihood solutions can be obtained using expectation-maximization or Gibbs sampling.

Our approach to clustering multi-tissue data combines ideas from existing ordinary (‘single-tissue’) and multi-species model-based clustering methods. We use the generative model of Qin (2006) and Joshi *et al.* (2008) to obtain the posterior probability for a (single-tissue) clustering given a (single-tissue) dataset. From Roy *et al.* (2013) we use the idea that a multi-tissue clustering consists of a set of *linked* clusters, where cluster *k* in one tissue corresponds to cluster *k* in any other tissue, and each cluster *k* contains a *core* set of genes, belonging to cluster *k* in *all* tissues, and a *differential* set of tissue-specific genes, belonging to cluster *k* in one or more, but not all, tissues. Like Roy *et al.* (2013), we assume that the data from one tissue can influence the clustering in another tissue, albeit via a simpler mechanism as we do not aim to reconstruct any phylogenetic histories among tissues. In brief, we assume that the posterior probability distribution of clusterings in tissue *t* is given by its ordinary single-tissue distribution given the expression data for tissue *t*, multiplied by a tempered distribution for observing that same clustering given the expression data for all other tissues t′≠t. The degree of tempering determines the degree of influence of one tissue on another, and can be used to model known prior relationships between tissues. For instance, we expect *a priori* that coexpression clusters will be more similar between vascular tissues, than between vascular and metabolic tissues.

### 2.2 Statistical model for single-tissue clustering

Our method is based on previous single-tissue, model-based clustering algorithms (Joshi *et al.*, 2008; Qin, 2006). In brief, for an expression data matrix $X\in R^{G\times N}$ for *G* genes and *N* samples, a clustering C is defined as a partition of the genes into *K* non-overlapping sets *C_k_*. We assume that the data points for the genes in each cluster and each sample are normally distributed around an unknown mean and unknown variance/precision. Given a clustering C and a set of means and precisions $\left(\mu_{\mathrm{kn}},\tau_{\mathrm{kn}}\right)$ for each cluster *k* and sample *n*, we obtain a distribution on expression data matrices as

$$p(X|C,\{\mu_{\mathrm{kn}},\tau_{\mathrm{kn}}\})=\prod_{k=1}^{K}\prod_{n=1}^{N}\prod_{g\in C_{k}}p(x_{\mathrm{gn}}|\mu_{\mathrm{kn}},\tau_{\mathrm{kn}}).$$

Assuming a uniform prior on the clusterings C and independent normal-gamma priors on the normal distribution parameters, we can use Bayes’ rule to find the marginal posterior probability of observing a clustering C given data X, upto a normalization constant:

(1) $$P(C|X)\propto \prod_{k=1}^{K}\prod_{n=1}^{N}\iint p(\mu ,\tau )\prod_{g\in C_{k}}p(x_{\mathrm{gn}}|\mu ,\tau )\;d\mu d\tau .$$

Note that we use a capital ‘*P*’ to indicate that this is a *discrete* distribution. p(μ,τ)=p(μ|τ)p(τ) is the normal-gamma prior, with

$$p(\mu |\tau )={\left(\frac{\lambda_{0}\tau }{2\pi }\right)}^{1/2}e^{-\frac{\lambda_{0}\tau }{2}{\left(\mu -\mu_{0}\right)}^{2}},\;p(\tau )=\frac{\beta_{0}^{\alpha_{0}}}{\Gamma (\alpha_{0})}\tau^{\alpha_{0}-1}e^{-\beta_{0}\tau },$$

$\alpha_{0},\beta_{0},\lambda_{0}>0$ and $-\infty <\mu_{0}<\infty$ being the parameters of the normal-gamma prior distribution. We use the values $\alpha_{0}=\beta_{0}=\lambda_{0}=0.1$ and $\mu_{0}=0.0$, resulting in a non-informative prior. The double integral in (1) can be solved exactly in terms of the sufficient statistics $T_{\mathrm{kl}}^{\left(\alpha \right)}=\sum_{i\in C_{k}}\sum_{n=1}^{N}x_{\mathrm{in}}^{\alpha }$ (α=0,1,2) for each cluster, see Joshi *et al.* (2008) for details.

For computational purposes, the decomposition of Eq. (1) into a product of independent factors, one for each cluster and sample, is important. We write the log-likelihood or Bayesian score accordingly as:

(2) $$S(C)=\text{log}\;P(C|X)=\sum_{k=1}^{K}\sum_{n=1}^{N}S_{\mathrm{kn}}.$$

### 2.3 Statistical model for multi-tissue clustering

Next, we assume that expression data $X=[X_{1}\in R^{G\times N_{1}},\ldots ,X_{T}\in R^{G\times N_{T}}]$ is available for *G* genes in *T* tissues, with *N_t_* samples in each tissue t∈{1,…,T}. We define a multi-tissue clustering as a collection of single-tissue clusterings $C=\{C_{1},\ldots ,C_{T}\}$, and assume that the probability of observing C given data X is given by

(3) $$\begin{array}{c} P(C|X)=P(C_{1},\ldots ,C_{T}|X_{1},\ldots ,X_{T})\\ =\frac{1}{Z}\prod_{t=1}^{T}\{P(C_{t}|X_{t})\prod_{t\prime \neq t}P{\left(C_{t}|X_{t\prime }\right)}^{\lambda_{t,t\prime }}\}, \end{array}$$

where *Z* is a normalization constant which we henceforth will ignore, each factor $P(C_{t}|X_{t}^{\prime })$ is a single-tissue posterior probability distribution defined in Eq. (1), and $\lambda_{t,t\prime }\in [0,1]$ is a set of hyper-parameters that define the prior tissue similarities; for notational convenience we define $\lambda_{t,t}=1$.

Note that $P(C_{t}|X_{t\prime })$ is a discrete distribution measuring how well clustering $C_{t}$ is supported by data $X_{t\prime }$. Raising a discrete distribution to a power less than 1 has the effect of making the distribution more uniform. Hence in Eq. (3), we are asking that clustering $C_{t}$ is supported predominantly by data $X_{t}$ from its own tissue, but also, albeit to a lesser extent depending on the values of $\lambda_{t,t\prime }$, by data from the other tissues.

Optimizing Eq. (3) across all multi-tissue clusterings is challenging. A considerable simplification is obtained if we constrain the problem to multi-tissue clusterings with the *same* number of clusters *K* in each tissue. Denoting by $I_{t}$ the set of samples/individuals in tissue *t* and by $N=\sum_{t=1}^{T}N_{t}$ the total number of samples, the decomposition in Eq. (2) allows to write:

(4) $$\begin{array}{c} \;\text{log}\;P(C|X)=\sum_{t=1}^{T}\sum_{t\prime =1}^{T}\lambda_{t,t\prime }\;\text{log}\;P(C_{t}|X_{t\prime })\\ =\sum_{t=1}^{T}\sum_{t\prime =1}^{T}\lambda_{t,t\prime }\sum_{k=1}^{K}\sum_{n\in I_{t\prime }}S_{\mathrm{kn}}^{\left(t\right)}\\ =\sum_{t=1}^{T}\sum_{k=1}^{K}\sum_{n=1}^{N}\gamma_{n}^{\left(t\right)}S_{\mathrm{kn}}^{\left(t\right)}, \end{array}$$

where we used $\lambda_{t,t}=1$, defined $\gamma_{n}^{\left(t\right)}\equiv \lambda_{t,t(n)}$, with *t*(*n*) the tissue to which sample *n* belongs, and wrote $S_{\mathrm{kn}}^{\left(t\right)}$ to denote the Bayesian score of clustering $C_{t}$ with respect to sample *n*.

Two extremal choices for the hyper-parameters are of interest. If $\lambda_{t,t\prime }=1$ for all t,t′, then the Bayesian score

(5) $$S^{\left(t\right)}=\sum_{k=1}^{K}\sum_{n=1}^{N}\gamma_{n}^{\left(t\right)}S_{\mathrm{kn}}^{\left(t\right)}$$

is the same for each tissue *t* and identical to Eq. (2) for the concatenated data matrix $X=[X_{1},\ldots ,X_{T}]$. Hence this is equivalent to clustering the entire dataset as if it came from a single-tissue (‘horizontal’ data concatenation). If $\lambda_{t,t\prime }=0$ for t′≠t, then Eq. (3) decomposes as a product of independent single-tissue factors. This is equivalent to clustering each tissue sub-dataset independently.

### 2.4 Optimization algorithm

To find a local maximum of the Bayesian score in Eq. (4), the following heuristic, greedy optimization algorithm was used:

1. **Data standardization:** Using appropriately normalized gene expression data, each gene is standardized to have mean zero and standard deviation one on the concatenated data X.
2. **Determine the number of clusters:** K-means clustering is run on the concatenated data with the number of clusters ranging from 2 to 100. The optimal number *K* is selected by visual inspection of an elbow plot.
3. **Initialize multi-tissue clustering:** Starting from the k-means clustering output at the selected number of clusters, genes are reassigned until a local optimum is reached for the single-tissue score Eq. (2) on the concatenated data X. All $C_{t}$ are initialized by this clustering.
4. **Optimize multi-tissue clustering:** For each tissue *t*, optimize $C_{t}$ by finding a local maximum for the Bayesian score Eq. (5) using single-gene reassignments; only gene reassignments improving the score by a minimum threshold *ϵ* are considered.

Note that even in the case $\lambda_{t,t\prime }=0$ for t′≠t, which removes all tissue dependencies in the Bayesian score (4), this algorithm still results in a multi-tissue clustering with linked clusters, due to each tissue being initialized by the same clustering and converging to a local optimum.

### 2.5 Implementation

The statistical model and optimization algorithm have been implemented in Java, as an extension of the ‘task’ revamp in the Lemon-Tree software (Bonnet *et al.*, 2015; Erola *et al.*, 2019), available at https://github.com/eb00/lemon-tree.

### 2.6 The Stockholm Atherosclerosis Gene Expression dataset

In the STockholm Atherosclerosis Gene Expression (STAGE) study, 612 tissue samples from 121 individuals were obtained during coronary artery bypass grafting surgery from the atherosclerotic arterial wall (AAW, *n *=* *73), internal mammary artery (IMA, *n *=* *88), liver (*n *=* *87), skeletal muscle (SM, *n *=* *89), subcutaneous fat (SF, *n *=* *72) and visceral fat (VF, *n *=* *98) of well-characterized CAD patients; fasting whole blood (WB) was obtained for isolation of DNA (*n *=* *109) and RNA (*n *=* *105) and biochemical analyses. Gene expression profiles from RNA samples of different tissues were jointly normalized to enable comparison across tissues (Foroughi Asl *et al.*, 2015; Hägg *et al.*, 2009; Talukdar *et al.*, 2016). 4956 genes with variance greater than 1 across all 612 samples were selected for further analysis, and subsequently standardized to have mean zero and standard deviation one, again across all 612 samples.

### 2.7 Multi-tissue clustering methods for comparison

We ran four multi-tissue clustering methods (see Supplementary Fig. S1):

- Revamp with reassignment threshold ϵ=0.005 and prior tissue similarities $\lambda_{t,t\prime }=\rho_{t,t\prime }^{\alpha }$, where $\rho_{t,t\prime }$ is the average correlation coefficient between samples from tissue *t* and t′ measured in the same individual and α=0.25 is a dissipation parameter to scale the correlation values. Here we suggest to derive the similarity coefficients using Pearson’s correlation, but other distance measures could be used.
- Revamp with reassignment threshold ϵ=0.005 and prior tissue similarities $\lambda_{t,t\prime }=0$.
- An alternative method, which treats the expression profile of each gene *g* in each tissue *t* as a separate (gene, tissue) variable and clusters the resulting (gene, tissue)-by-individual expression matrix using the single-tissue clustering algorithm (Section 2.2). This results in a single set of clusters, which are disentangled into a set of linked clusters, by assigning gene *g* to cluster *m* in tissue *t* whenever (*g*, *t*) belongs to original cluster *m*. This method was called ‘vertical data concatenation’ before, and relies on having expression data from multiple tissues in the *same* individual. In STAGE, 21 individuals had data in all 7 tissues.
- Single-tissue clustering on the entire dataset of 612 samples (called ‘horizontal data concatenation’ before). This results in an identical clustering across all tissues. It is not a true multi-tissue clustering method, but is used as an overall benchmark to determine the relevance of a multi-tissue approach.

### 2.8 Validation data

To evaluate the biological relevance of each multi-tissue clustering method, we used the following approach:

- We performed GSEA using first the GOSlim ontology, that gives a broad overview of the ontology content without the detail of the specific fine-grained terms (http://www.geneontology.org/page/go-slim-and-subset-guide), and after on GO terms (http://www.geneontology.org/page/download-ontology).
- We assigned sets of ‘regulators’ to each of the modules considering as candidate regulators the tissue-specific sets of genes with significant eQTLs identified in Foroughi Asl *et al.* (2015) (2464 AAW, 3209 IMA, 4491 liver, 2534 SM, 2373 SF, 2994 VF and 5691 WB genes).
- We obtained human tissue protein–protein interaction (PPI) networks from Barshir *et al.* (2013). Specifically, we used TissueNet v2 networks consisting of curated experimentally detected PPIs between proteins expressed in Genotype-Tissue Expression dataset tissues ‘Artery Aorta’, ‘Liver’, ‘Muscle Skeletal’, ‘Adipose Subcutaneous’, ‘Adipose Visceral’ and ‘Whole Blood’, available for download at http://netbio.bgu.ac.il/labwebsite/? q=tissuenet2-download.

### 2.9 Validation methods

We tested for GO functional enrichment using the task go_annotation in the Lemon-Tree software, and task regulators were used to identify gene ‘regulators’ using a probabilistic scoring (Joshi *et al.*, 2009).

To test for enrichment of known PPIs in a given clustering, we calculated the fold-change enrichment as

$$\text{FC}=\frac{\frac{\text{Number}\;\text{of}\;\text{co}-\text{clustered}\;\text{gene}\;\text{pairs}\;\text{with}\;\text{PPI}}{\text{Total}\;\text{number}\;\text{of}\;\text{PPI}}}{\frac{\text{Total}\;\text{number}\;\text{of}\;\text{co}-\text{clustered}\;\text{gene}\;\text{pairs}}{\text{Total}\;\text{number}\;\text{of}\;\text{gene}\;\text{pairs}}}\;\;.$$

All clustering methods were run on the seven available STAGE tissues, and the results for six tissues were used for validation (IMA did not have a matching tissue in the TissueNet database). To evaluate the clustering of a particular tissue, we used all PPIs for that tissue. To evaluate the core gene set of a cluster (for cluster *m*, the set of genes belonging to *m* in all tissues), we used the set of PPIs shared across all tissues.

Because the fold-change value is influenced by the number of clusters (more clusters results in fewer co-clustered pairs), we used the same number (*k *=* *12) of clusters for all compared methods (Section 2.7).

## 3 Results

### 3.1 Multi-tissue clustering with revamp produces mappable clusters with tunable overlap levels

To identify co-expression clusters that reflect biological similarities and differences across tissues, we analyzed samples from seven tissues from the STAGE study. First we initialized revamp with the partition obtained from clustering all tissue samples using k-means with *k *=* *12 clusters for all our analyses, as this value was near the inflection point of the elbow plots in all tissues (Supplementary Fig. S2). Then we updated the cluster assignments for each tissue independently using our Bayesian model-based score that depends on a set of hyper-parameters $\lambda_{t,t\prime }$, expressing prior beliefs on pairwise tissue similarities (Section 2.3), using a greedy optimization algorithm that has one free parameter *ϵ*, the minimum gain in Bayesian score for reassigning a gene from one cluster to another (Section 2.4). The resulting multi-tissue clustering consists of a set of linked clusters, where cluster *k* in one tissue corresponds to cluster *k* in any other tissue. Genes that belong to a particular cluster *k* in *all* tissues form a core set of genes with conserved coexpression across tissues, whereas genes that belong to cluster *k* in one or more, but not all, tissues form tissue-specific sets of genes that are *differentially* coexpressed with the core of cluster *k*.

To test the influence of the method parameters, we systematically tested a large space of parameter combinations (Supplementary Fig. S3). Both the reassignment threshold *ϵ* and tissue similarities $\lambda_{t,t\prime }$ ultimately govern the degree of overlap across tissues of the linked clusters, with small thresholds and near-zero similarities leading to nearly tissue-independent clusterings, and large thresholds and/or near-one similarities leading to nearly identical clusterings. Although *ϵ* and $\lambda_{t,t\prime }$ are to some extent interchangeable (i.e. a smaller threshold value can be compensated by a uniform increase in similarity values), setting *ϵ* to a small, non-zero value is recommended to avoid spurious reassignments due to numerical round-off errors in the Bayesian score calculation.

When comparing this partitioning with clustering tissues independently, the cluster quality is improved (Supplementary Table S1) and the similarities between tissues are stronger. The functional enrichment analysis revealed that a larger proportion of functional enriched categories were shared across two or more tissues (Supplementary Fig. S4). Moreover, similarity heatmaps showed that the degree of shared enrichment between tissues in our clustering was able to reflect the degree of overall expression similarity (Supplementary Fig. S5). Yet it is noteworthy to mention that multi-tissue clustering methods, and in particular revamp when using prior tissue similarities that is optimized based on Eq. (5), may show fuzzy borders when assessed with traditional validation methods like silhouette scores (see Supplementary Fig. S6).

### 3.2 Revamp multi-tissue clustering is more enriched for tissue protein–protein interactions than other approaches

To evaluate the performance of revamp, we ran four different multi-tissue clustering methods (see Methods), testing for each one for the enrichment of human tissue protein-protein interactions (PPIs) from the TissueNet database (Barshir *et al.*, 2013) among co-clustered genes, using six tissues that matched between STAGE and TissueNet.

**Fig. 1.**
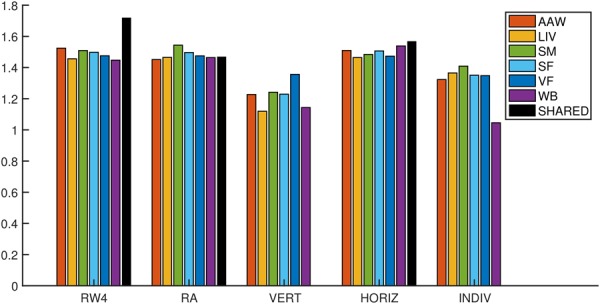
Fold-change enrichment of tissue PPIs in tissue clusters for four multi-tissue clustering methods and individual single-tissue clustering. RW4—revamp with prior tissue similarities set according to their overall expression correlation, RA—revamp with prior tissue similarities set to zero, VERT—vertical data concatenation, HORIZ—horizontal data concatenation, INDIV—each tissue clustered individually. Each colored bar shows the fold-change overlap of tissue PPIs in clusters for the matching tissue; the black bar shows the fold-change overlap of tissue-shared PPIs in tissue-shared genes of linked clusters. See Section 2 for details. (Color version of this figure is available at *Bioinformatics* online.)

On a tissue-by-tissue basis, running revamp with or without prior tissue similarity values resulted in similar fold-change enrichment values for tissue PPIs (average fold-change over 6 tissues of 1.49 and 1.48, respectively) as running single-tissue clustering on all samples together (average fold-change 1.50), and considerably higher enrichment than using vertically concatenated data (average fold-change 1.22) (Fig. 1). For a baseline reference, we also calculated enrichment for each tissue clustered individually using the single-tissue clustering method. Consistent with the assumption that analyzing data integratively using multi-tissue clustering should improve biological relevance, single-tissue clustering resulted in lower fold-change values (average fold-change 1.31) (Fig. 1).

We further reasoned that genes assigned consistently to the same cluster across all tissues (‘core’ cluster genes) should reflect tissue-independent interactions between these genes. To test this hypothesis, we calculated enrichment of tissue-independent PPIs (i.e. PPIs present in all six tissue PPI networks) among core cluster genes. For revamp with prior tissue similarity values, a significant increase in enrichment for tissue-independent PPIs was observed (fold-change 1.72), whereas for revamp without prior tissue similarities and horizontal data concatenation no difference was observed compared to all tissue PPIs (fold-changes 1.47 and 1.57, respectively) (Fig. 1). Vertical data concatenation resulted in very small core gene sets, containing no known tissue-independent PPIs (see also Supplementary Table S2).

### 3.3 Functional predictions by Revamp clusters and gene regulators associated with CAD

To test whether the clustering algorithm accurately captures the higher-level biological process represented by each module we first performed gene ontology enrichment analysis (see top enrichments in Supplementary Table S3). Network analysis revealed three connected components: clusters 5, 9 and 10 were related with immune system response; the lipid metabolic process was enriched in clusters 4, 6 and 7; and clusters 0 and 8 were associated with cell adhesion and extracellular matrix organization.

**Fig. 2.**
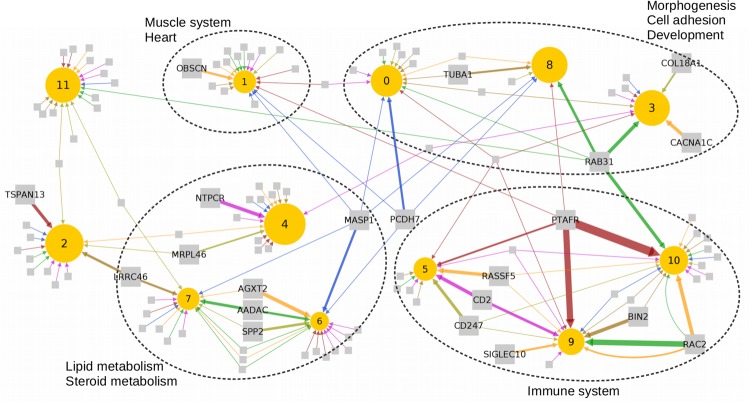
Module regulatory network for all seven tissues. Regulators are presented as squares and clusters as circles with size proportional to the number of genes in the cluster. Only the regulators with a score greater than 20 in the regulators task are represented, and we named those with a score above 60. Edges are colored per tissue as per Figure 3, and their width is proportional to the regulator score. (Color version of this figure is available at *Bioinformatics* online.)

Then we ran independently on each tissue the regulator probabilistic scoring task (see Section 2.9) to predict upstream regulatory genes, considering as candidate regulators the tissue-specific genes with genetic variants in their regulatory regions affecting gene expression (‘*cis*-eQTL effects’). The regulatory network of the most significant regulators for the inferred modules is depicted in Figure 2.

The development of atherosclerosis is in large part mediated by the inflammatory cascade (Crowther, 2005). Our results indicated that the inflammatory response in AAW may be regulated by PTAFR, a mediator in platelet aggregation and the inflammatory response (Perisic *et al.*, 2016; Rastogi *et al.*, 2008). SF and VF were shown to be regulated by SIGLEC10 and CD247, respectively, genes that have been previously associated with CAD (Ammirati *et al.*, 2008; Shen *et al.*, 2013). Other tissues were linked to the previously identified inflammatory regulators BIN2 (Liao *et al.*, 2011), CD2 (Hansson and Libby, 2006), RAC2, that also directs plaque osteogenesis (Ceneri *et al.*, 2017), and the pro-apoptotic regulator of RAS protein, RASSF5 (Dejeans *et al.*, 2010).

Lipid metabolism also plays a key role in the development of atheroma plaques. Metabolism-related clusters 6 and 7 were found to be regulated by AGXT2 and SPP2, in SF and VF respectively. AGXT2 polymorphisms were identified as risk for CAD in Asian populations (Yoshino *et al.*, 2014; Zhou *et al.*, 2014), and SPP2 may contribute to the atheroprotective effects of HDL (Abdel-Latif *et al.*, 2015). AADAC, that controls the export of sterols (Tiwari *et al.*, 2007), may also be a regulator in SM. In WB, we found MASP1, a gene associated with a decreased lectin pathway activity in acute myocardial infarction patients (Yan *et al.*, 2016).

The atherogenic pathway involves the inflammation of the arterial wall, injury of the intima, lipid infiltration and activation of the angiogenic signaling, processes that involve a dysfunction in the cell adhesion (Sun, 2014). Our analysis showed that RAB31, which induces lipid accumulation in atheroma plaques (Fu *et al.*, 2002), regulates the morphogenesis-related clusters 3 and 8 in SM. Cluster 3 was also shown to be regulated by CACNA1C in SF, a gene involved in calcium channels and associated with inherited cardiac arrhythmia (Kawashiri *et al.*, 2014), and COL18A1 in VF, that may control angiogenesis and vascular permeability (Moulton *et al.*, 1999). The expression levels of PCDH7, gene involved in cell adhesion, and TUBA1 were also previously correlated with CAD (Chittur *et al.*, 2008; Eyster *et al.*, 2011; Sinnaeve *et al.*, 2009).

### 3.4 Revamp discovers multi-tissue clusters underlying CAD phenotypes

**Fig. 3.**
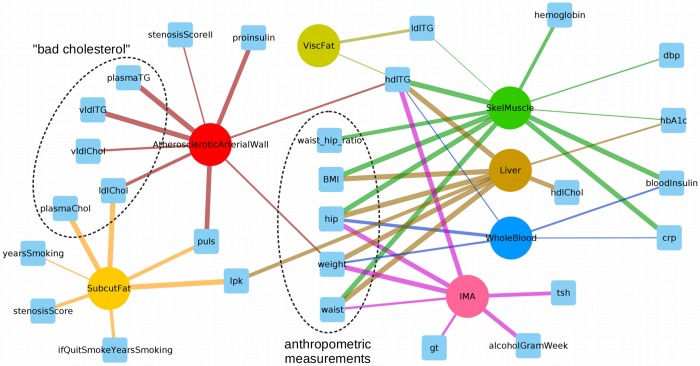
Network representation of the correlation between the eigengenes, the first principal component of a given module, and relevant CAD phenotypes (squares), aggregated per tissue (circles). Edge width is inversely proportional to the correlation *P*-value

The systems genetics paradigm says that genetic variants in regulatory regions affect nearby gene expression (‘*cis*-eQTL effects’), which then causes variation in downstream gene networks (‘*trans*-eQTL effects’) and clinical phenotypes. Ultimately, gene-gene interactions across metabolic and vascular tissues will enable information flow to the end stage phenotypic changes in CAD. We therefore used regression analysis to identify associations between module gene expression and CAD phenotypes (see Talukdar *et al.*, 2016), as presented in Figure 3.

The aggregated results revealed that AAW and SF are the main tissues associated with very-low-density lipoprotein (VLDL) and low-density lipoprotein (LDL) cholesterol levels, while the liver was the main tissue associated with high-density lipoprotein (HDL) cholesterol. Fat has been previously identified as the main contributor of CAD heritability, and the top regulatory networks in CAD have shown to be strongly enriched in associations with plasma levels of HDL, LDL and pro-insulin (Zeng *et al.*, 2019), as it is depicted in the left part of Figure 3.

Besides that, IMA was found to be associated in cluster 3 with the thyroid-stimulating hormone, that causes many hemodynamic effects and influences the structure of the heart and circulatory system (Grais and Sowers, 2014), and alcohol consumption in clusters 5 and 9, whose associations with cardiovascular diseases are heterogeneous (Bell *et al.*, 2017).

On the other hand, the results showed that the phenotypes related to anthropometric measurements are mostly associated with SM, liver and IMA, and with less significance with WB and AAW, but not with SF and VF. If we focus on clusters related to body weight, as a typical example of a trait regulated by, and affecting multiple tissues, we can find gene regulators such as PTAFR (in AAW) and CD2 (in IMA) which have been described to affect food intake and body weight, apart from the inflammatory response (Are Hanssen *et al.*, 2004; Li and McIntyre, 2015). In SM, RAC31 may influence on the body weight by mediating the insulin-stimulated glucose uptake (Lyons *et al.*, 1999). Last, also the candidate regulators BIN2 and RAC2 have been associated with obesity and metabolic syndrome (Aguilera *et al.*, 2013; Zhang *et al.*, 2005).

## 4 Conclusion

Herein we proposed a Bayesian model-based multi-tissue clustering algorithm, revamp, which incorporates prior information on physiological tissue similarity, and which results in a set of clusters consisting of a core set of genes conserved across tissues as well as differential sets of genes specific to one or more subsets of tissues. Using data from seven vascular and metabolic tissues from the STAGE study, we demonstrated that our method resulted in multi-tissue clusters with higher enrichment of tissue-specific protein-protein interactions than comparable clustering algorithms. Moreover, the multi-tissue clusters highlighted the ability of revamp to link together regulatory genes, biological processes and clinical patient characteristics in a meaningful way across multiple tissues, and we believe this makes it an attractive and statistically sound method for analyzing multi-tissue gene expression datatsets in general. Revamp is implemented and freely available in the Lemon-Tree software at https://github.com/eb00/lemon-tree.

## Funding

This work was supported by BBSRC [Roslin Institute Strategic Programme, BB/P013732/1] and the NIH [NHLBI R01HL125863]. P.E. has been partially supported by CRUK [C18281/A19169].

*Conflict of Interest*: none declared.

## Supplementary Material

btz805_Supplementary_Data
