# Supplementary material for: Model-based clustering of multi-tissue gene expression data
Source: Bioinformatics. 2019 Nov 5;36(6):1807–13. doi: 10.1093/bioinformatics/btz805 (PMC7162352; doi:10.1093/bioinformatics/btz805)
Supplement: btz805_Supplementary_Data [file btz805_supplementary_data.pdf]

Supplementary Material: Model-based clustering of multi-tissue gene expression data

Pau Erola [1,2], Johan LM Björkegren [3,4], and Tom Michoel [1,5]

[1]Division of Genetics and Genomics, The Roslin Institute, The University of Edinburgh, Midlothian EH25 9RG, Scotland, United Kingdom. [2]MRC Integrative Epidemiology Unit, University of Bristol, Bristol BS8 2BN, United Kingdom. [3]Department of Genetics and Genomic Sciences, Institute of Genomics and Multiscale Biology, Icahn School of Medicine at Mount Sinai, NY 10029, New York, USA. [4]Integrated Cardio Metabolic Centre (ICMC), Karolinska Institutet, 141 57, Huddinge, Sweden. [5]Computational Biology Unit, Department of Informatics, University of Bergen, N-5020 Bergen, Norway.

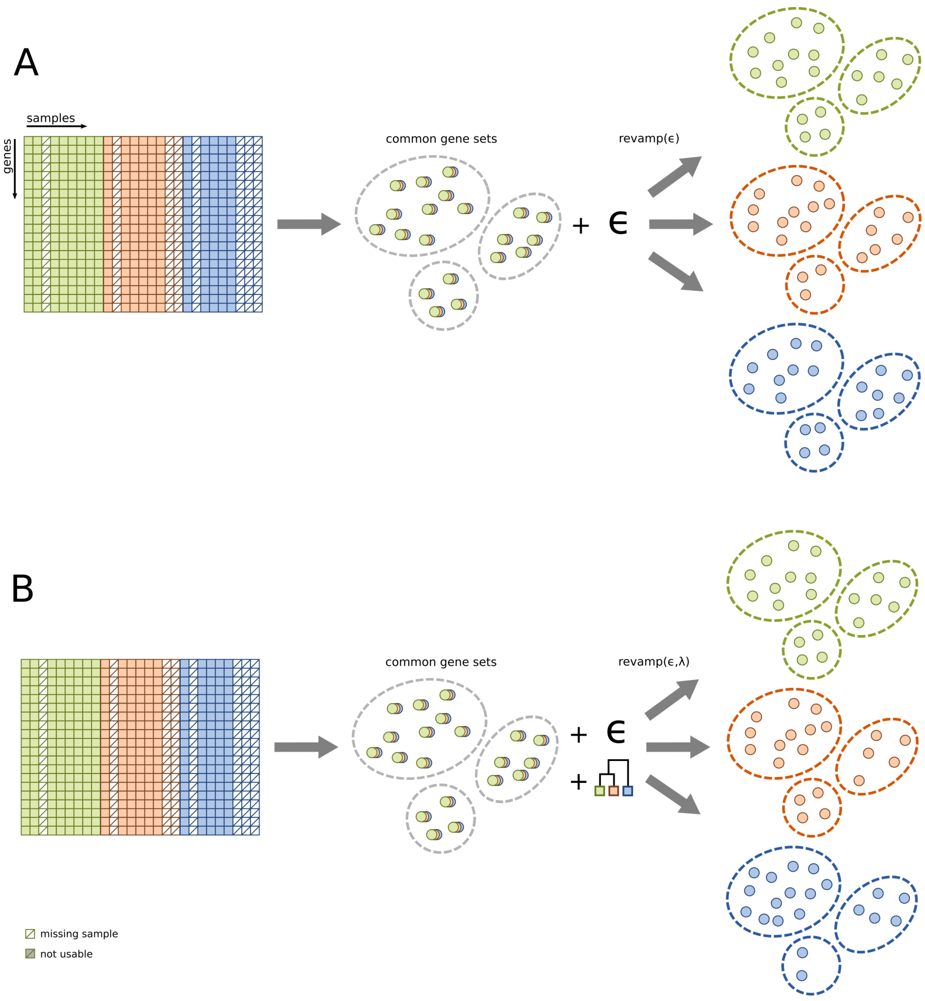

Supplemental Fig. 1: Visual description of the four multi-tissue clustering methods used for comparison. (A) `Revamp` method initializes the common clustering on the concatenated data and after optimizes the multi-tissue clustering by reassigning genes that improve the Bayesian score more than the threshold parameter ϵ. (B) `Revamp` with reassignment threshold ϵ and prior tissue similarities $\lambda_{t,t'}$.

C

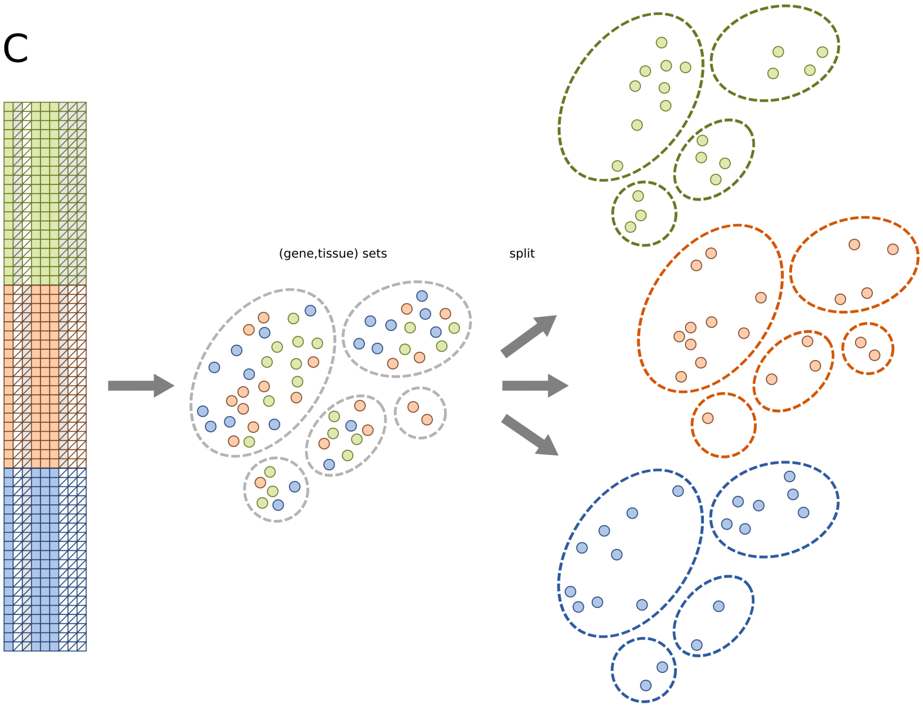

D

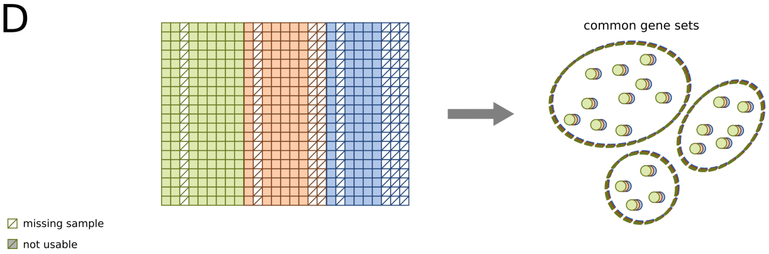

Supplemental Fig. 1 (Cont.): (C) Vertical data concatenation considers the expression profile of each gene g in each tissue t as a separate (gene, tissue) variable. The result is a single set of clusters that we can split for each tissue. (D) As an overall benchmark, we used a single-tissue clustering on the horizontally concatenated dataset, which will result in identical partitions across all tissues.

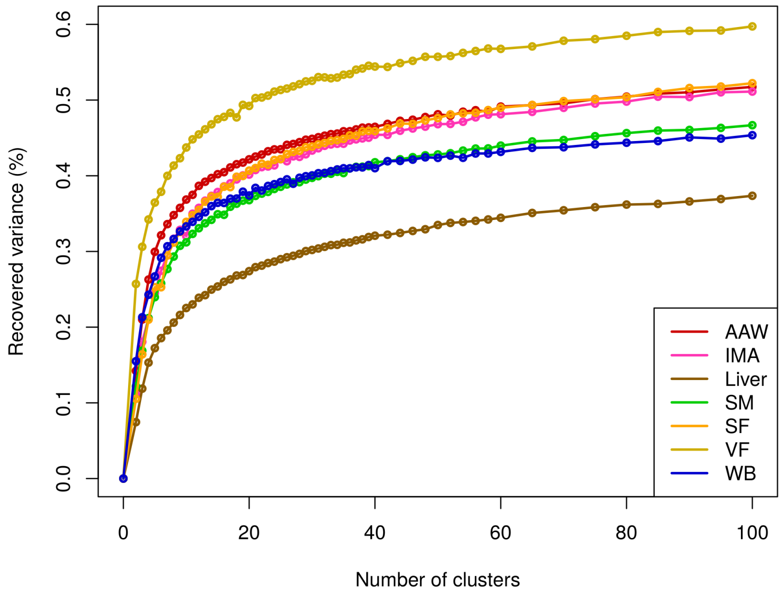

Supplemental Fig. 2: Elbow plot showing the percentage of variance explained as a function of the number of clusters ($k = 2, 3, ...100$) in the partitions obtained with k-means algorithm. The inflection point of the curve in all tissues is near $k = 12$ clusters.

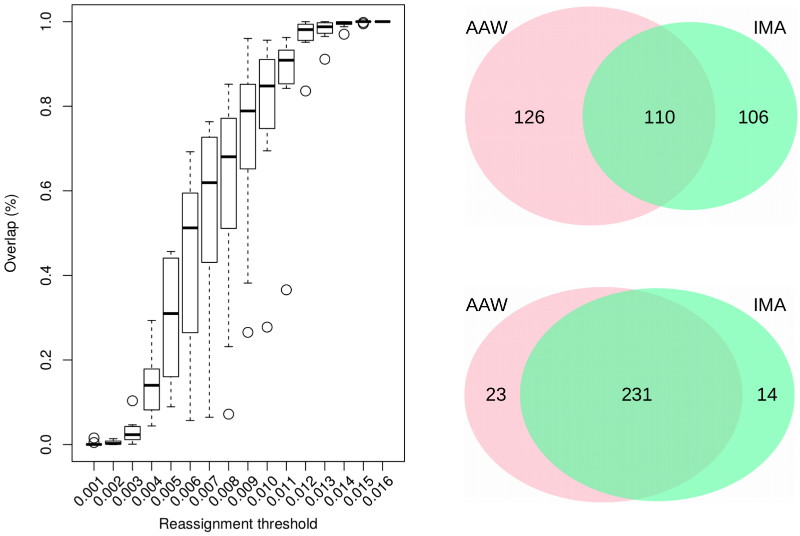

Supplemental Fig. 3: We systematically tested a large space of parameter combinations for ϵ and λ. On the left, average percentage of genes that overlap in each cluster as function of the reassignment parameter ϵ. Although an optimal point cannot be fixed, it is important to choose a threshold that makes the comparison of clusters meaningful, i.e. the core set of genes preserved across tissues is significant. On the right, two Venn diagrams that compare the set of genes of one cluster between two tissues, AAW and IMA. The number of common genes is greater when using the threshold parameter $\epsilon = 0.005$ (bottom), than when $\epsilon = 0.001$ (top).

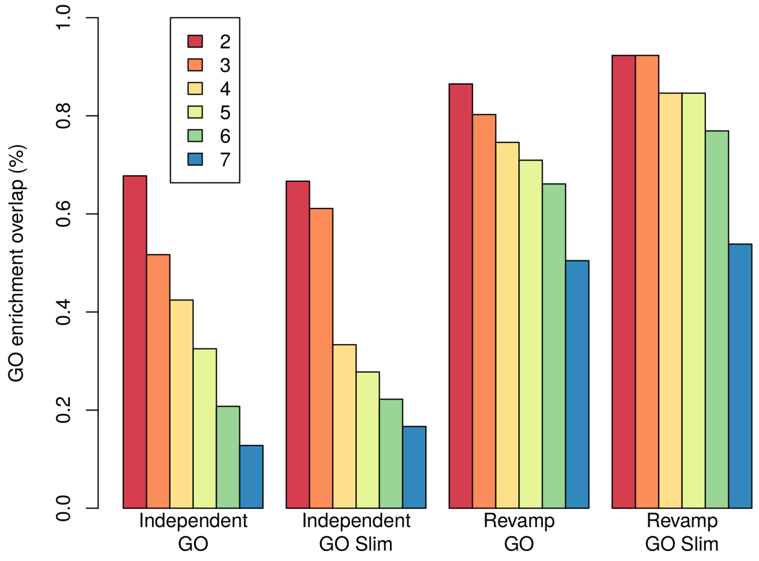

Supplemental Fig. 4: Percentage of shared enriched GO terms between different number of tissues for independent clustering and the proposed multi-tissue clustering method.

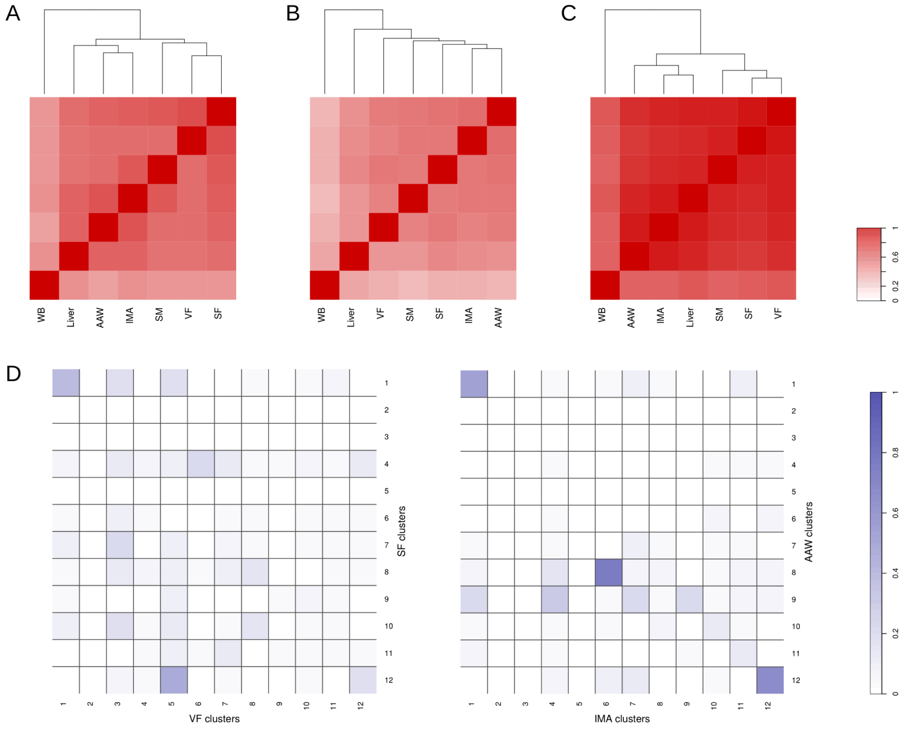

Supplemental Fig. 5: On the top, the correlation matrix of gene expression values between tissues (A) and the matrix of shared GO enrichments between tissues obtained with clustering the tissues independently (B) and with our method `revamp` (C). On the bottom, percentage of genes that are present in the same cluster both adipose (SF, VF) and vascular (AAW, IMA) tissues after clustering tissues independently (D).

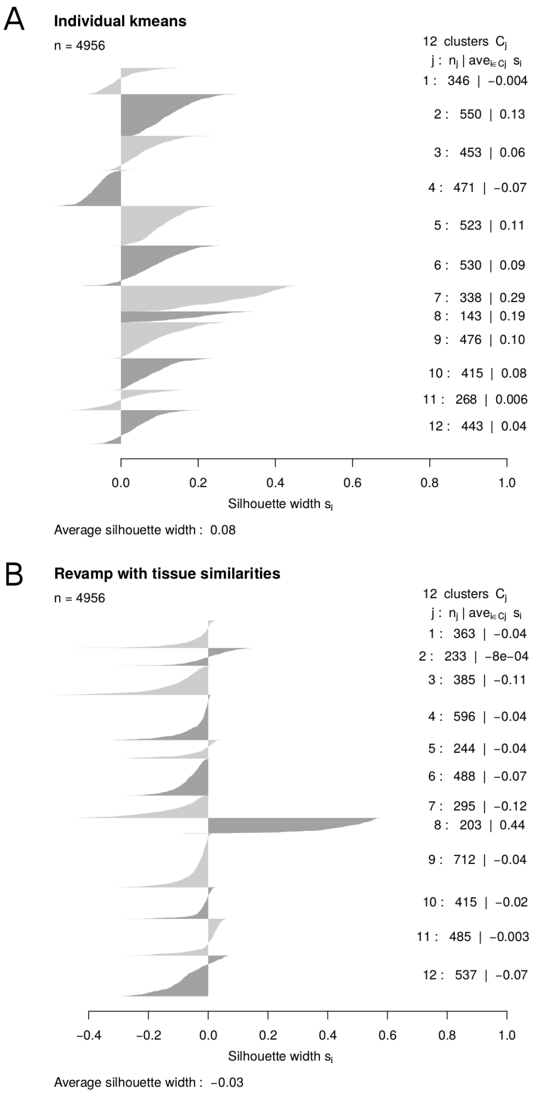

Supplemental Fig. 6: Silhouette plots (using euclidian distance) of partitioning AAW samples using (A) single-tissue K-means method and (B) `revamp` with prior tissue similaritites. Silhouette score may give the false impression that our method performs worse, but in fact the measure is based on pairwise distances and hence favours methods that optimize pairwise distance scores. On the other side, multi-tissue clustering methods like `revamp` optimize the partitioning to fit all tissues, potentially creating more fuzzy clusters when assessed by distance. Indeed, the clustering from panel (A) has a much lower Bayesian score than the clustering from panel (B) as it is shown in the first row of Supplemental Table 1, first and last columns.

	independent		multi-tissue		
	**k-means**	**RA**	**k-means**	**RA**	**RW4**
AAW	-761.88	-772.26	-582.46	-594.31	-567.74
IMA	-756.44	-771.08	-606.28	-589.88	-569.90
Liver	-763.81	-772.70	-564.46	-566.84	-552.27
Skeletal Muscle	-730.91	-760.69	-585.24	-622.95	-572.07
Subcutaneous Fat	-810.40	-813.19	-598.88	-597.46	-581.64
Visceral Fat	-778.14	-783.25	-575.23	-688.63	-564.77
Whole Blood	-591.50	-608.92	-457.31	-498.31	-427.70

Supplemental Table 1: Final network score per gene for different clustering methods; greater values are better. For each tissue we computed the Bayesian score eq. (5) of each partition on the whole dataset, and considering the average correlation coefficient between tissues as prior inter-tissue similarity. Clustering tissues independently has a bad fit to the model, while multi-tissue algorithms like kmeans (horizontal concatenation) and RA ignore the constrains between tissues resulting in lower scores.

	vertical	revamp (RW4)	
		Subset	All samples
Atherosclerotic Arterial Wall	-824.14	-753.05	-567.74
Internal Mammary Artery	-813.79	-767.71	-569.90
Liver	-800.80	-739.93	-552.27
Skeletal Muscle	-794.88	-742.15	-572.07
Subcutaneous Fat	-816.05	-773.34	-581.64
Visceral Fat	-755.61	-746.94	-564.77
Whole Blood	-636.87	-561.61	-427.70

Supplemental Table 2: Final network score per gene for vertical clustering (VERT) and `revamp` using only the subset of 21 complete samples, and the whole dataset; greater values are better. The first two approaches have worse scores in part because of the substantially smaller training data.

GO term	cluster	p-val range
immune system process	5,9,10	1.04E-19 – 3.00E-5
signal transduction	9	1.28E-2 – 3.82E-2
macromolecular complex assembly	10	1.13E-2 – 4.88E-2
carbohydrate metabolic process	10	1.40E-2 – 3.82E-2
protein complex assembly	10	1.22E-2 – 4.88E-2
cellular component assembly	10	1.22E-2 – 4.88E-2
lipid metabolic process	4,6,7	6.26E-4 – 1.89E-2
transmembrane transport	6	2.98E-3 – 2.98E-3
cell adhesion	0,8	2.54E-9 – 9.57E-5
extracellular matrix organization	0,8	6.24E-5 – 1.84E-2
cell proliferation	0	2.73E-3 – 2.54E-2
cell-cell signaling	8	2.95E-3 – 3.33E-2

Supplemental Table 3: GO functional enrichment using GOSlim ontology. The top terms presented are associated with all seven STAGE tissues.
